# Peptide-based recycling of critical raw materials from electronic waste: The transition towards a circular economy requires novel and environmentally-friendly solutions to increase the recycling rate from discarded electronic devices

**DOI:** 10.1038/s44319-025-00449-x

**Published:** 2025-04-23

**Authors:** Franziska L Lederer, Peter Boelens

**Affiliations:** Biotechnology Department, Helmholtz Institute Freiberg for Resource Technology, Helmholtz-Zentrum Dresden-Rossendorf, Dresden, Germany

**Keywords:** Biotechnology & Synthetic Biology, Economics, Law & Politics

## Abstract

Biology-based tools, notably metal-binding peptides, could help to considerably increase the recycling rate of metals in electronic waste as required by EU law and for a more sustainable economy.

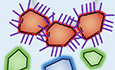

Unfettered economic growth and exploitation of natural resources has had a devastating impact on climate, biodiversity and ecosystems and puts pressure on states to adopt policies and other measures to support more sustainable development. One essential component of sustainability is a circular economy that recycles used materials from no-longer-used products to replace a linear economy that extracts non-renewable resources from the planet to produce products that eventually end up in landfills and incinerators. In this regard, the electronics industry, as a major consumer of a wide range of non-renewable resources, has a great potential for recycling essential components and elements from discarded products. The amount of electronic products produced and discarded is increasing constantly. In 2022, 14.4 million tonnes of electronic devices were produced in the EU and 5.5 million tonnes of these ended up as waste. In addition to plastic housings, recyclable elements include common metals such as silver, copper or aluminum, as well as critical raw materials including lithium, gallium, cobalt and the rare-earth elements europium, erbium or neodymium that are used in batteries, lighting, semiconductors, lasers, capacitors or fiber optics amongst many others.

“… the electronics industry, as a major consumer of a wide range of non-renewable resources, has a great potential for recycling essential components and elements from discarded products.”

Unlike their name implies, rare-earth elements are not rare but occur only in low concentrations in the Earth crust and have to be extracted from vast amounts of raw ore to obtain sufficient quantities. Their mining, purification, and processing is therefore complex, consume enormous amounts of energy, and cause serious environmental problems as well as health problems for miners and workers. At the same time, the global demand for these elements has been increasing at a rapid pace owing to population growth, increasing digitalization and a greater demand for electronic devices of all kinds. Moreover, electronic devices have an increasing complexity in terms of the number of different metals used in various compounds.

## Legal requirements to increase recycling rates

At the end of a high-tech product’s lifetime, only the two or three most valuable elements are conventionally recycled, while the majority of elements are discarded. To achieve a sustainable, circular economy, we need novel recycling strategies to efficiently and economically recover most if not all elements from an electronic device. Such approaches must also integrate novel tools with conventional recycling methods to maximize the extraction and reuse of valuable elements from electronic waste.

Current recycling rates of most economically important metals remain poor; at the same time, the EU faces a supply risk of critical raw materials such as the platinum group metals (PGMs) and rare-earth elements (REEs) (Table 1). Although the recycling industry is very efficient in processing e-waste and recovering most valuable elements, the need for further improvement and extension to other critical raw materials out of electronic end-of-life products (EoL) comes from legislative instruments, such as the EU’s Critical Raw Materials Act (CRMA). This legislation sets a benchmark that, by 2030, at least 25% of the EU’s annual consumption of critical raw materials must come from recycling. In addition, producers of electronic devices that contain permanent magnets will be required to label their products with information on the size, weight, and chemical composition of the magnets used as well as on additives used. Producers of electronic devices are encouraged to use recycled material for new devices.

**Table 1:** Applications, economic importance and end-of-life recycling input rates (EoL-RIR), main supplying countries and supply risk of selected metals based on Grohol and Veeh, 2023.

Metal	Main applications	Economic importance	Eol- RIR (%)	Main EU/global suppliers	Supply risk
PGM	Autocatalysis, jewelry, chemical manufacture	7.1	12	NA^c^	2.7
Lithium	Batteries, glass and ceramics, steel and aluminum	3.9	0	Chile/China	1.9
Light/ heavy REE	Permanent magnets, lighting phosphors, catalysts	5.9/ 7.8	1	China	3.7/ 5.6
Gallium	Integrated circuits, lighting, CIGS solar cells	3.7	0	China	3.9
Copper^a^	Electrical infrastructure	4	55	Poland/Chile	0.1
Nickel^a^	Batteries, steel making, automotive	5.6	16	Russia/China	0.5
Gold^b^	Jewelry (85%), electronics (13%)	2.4	5		0.4
Silver^b^	Jewelry (24%), photovoltaics (14%), automotive (8%)	4.6	4		0.8

The metals are categorized as critical raw materials under the condition that Economic Importance >1.0 and Supply Risk >1.0.

^a^Cu and Ni do not meet the criteria for critical raw materials but are considered as strategic raw materials due to their importance for green and digital transition, defense and aerospace.

^b^Au and Ag are precious metals that do not meet the Supply Risk criteria. They are included in the list because their global value represent 16% and 1% of the global value of metals from e-waste.

^c^Main EU supplier is not available (NA). The main global supplier is South Africa for Ir, Pl, Rh, Ru, and Russia for Pd.

“Current recycling rates of most economically important metals remain poor; at the same time, the EU faces a supply risk of critical raw materials…”

Hence, the recycling industry faces critical challenges to integrate novel and more sustainable tools into its existing systems as traditional recycling facilities will not be able to meet the regulatory requirements with the current processes in place. The problems are complex and solutions are still in development.

## Biological approaches to recycling

Current recycling processes depend on the waste stream and the recycler. Generally, recycling processes for waste electrical and electronic equipment (WEEE) have the following steps in common: Preparation including collection, transport and presorting: pretreatment including dismantling and depollution from hazardous substances: processing to separation individual parts; and extraction and recovery of metals (Werner et al, 2020). In 2022, 46.31% of all WEEE in the EU and the USA were recycled. In 2022, according to the global e-waste monitor, all e-waste worldwide contained a total of 31 million tons of metals. In total, 12,000 tons of the metals in worldwide e-waste were REE. Classical recycling processes recovered 19 million tons of these metals, particularly copper, which makes up 2 million tons of e-waste and other precious metals such as gold (270 tons in e-waste) and silver (1200 tons in e-waste). However, recycling quotes for REE are still 1% or less owing to the low prices for raw materials, material complexity, particle size, insufficient economics and lack of sustainable recycling routes.

Thus, there is an increasing need for novel and more sustainable recycling tools that can extract higher amounts of a wider range of elements from WEEE. Biological recycling tools, which are compatible with established traditional recycling methods hold great promise to achieve this goal. These tools can contribute to increasing the amount of extracted elements, decreasing the environmental impact of toxic extraction agents as well as decreasing the amount of waste. Biological tools can facilitate the preprocessing of shredded e-waste, for instance, for the separation of aluminum in battery recycling. Finally, bioleaching, biosorption, and bioflotation can deal with waste that poses greater challenges for recycling: waste that is strongly diluted, contains nearly identical components, has complex mixtures, and so on (Pollmann et al, 2018; Zhuang et al, 2015). In fact, industrial-size biotechnological extraction methods are rapidly advancing. Approximately 15% of the worldwide copper and 5% of the worldwide gold production is already achieved with bioleaching (Brierley and Brierley, 2013).

Biomolecules have great potential as freely dissolved or immobilized recycling agents as they interact with high-affinity and specificity with their target material. Nature already provides a vast toolbox of metal-interacting biomolecules. Organic acids produced by bacteria and fungi are able to complex and leach metal ions out of primary (ore minerals) and secondary (e-waste) materials. Siderophores can complex trivalent metal ions such as gallium, indium or germanium with very high-affinity. Biosurfactants bind to metal ions with their hydrophilic head.

“Biomolecules have great potential as freely dissolved or immobilized recycling agents as they interact with high-affinity and specificity with their target material.”

Yet, no naturally occurring biomolecules with high-affinity and selectivity are known so far to bind to various critical elements of interest. To efficiently recycle these elements, artificial biomolecules are needed. Peptides are highly promising candidates for this purpose. They are typically non-toxic and biodegradable and can be produced from renewable resources, such as genetically recombinant plants or bacteria, with minimal to no environmental pollution and a negligible land use. Owing to their short chains, peptides are significantly more robust under industrial conditions—varying temperatures, pressures or pH levels—than proteins.

## Phage surface display to identify material binding peptides

Artificial biomolecules with desired affinities can be identified using different peptide-expressing tools, such as cell-surface display. We prefer the phage surface display (PSD) technology, which was first described in 1985 (Smith, 1985); its inventor, George P. Smith, was awarded with the Nobel Prize in Chemistry in 2018. The PSD technology uses libraries of filamentous bacteriophages (fd, M13) to express random peptide sequences. In the so-called biopanning process, the phage library is screened for phages that interact with high-affinity with the target material. This powerful tool was applied in many different fields, for instance, for investigating protein-protein interactions, identifying antibodies against cancer cells (Istomina et al, 2024) or for the nucleation of nanoparticles (Flynn et al, 2003). Since 2013, our BioKollekt group has been using PSD to identify artificial peptides for recycling purposes. Selectively binding peptides were found amongst others for LaPO_4_:Ce,Tb, Y_2_O_3_, and gallium ions (Lederer et al, 2017; Maass et al, 2024; Schönberger et al, 2019). There are numerous variables in PSD, including the size, charge and surface characteristics of the target material. In addition, the binding buffer, pH and elution medium can be varied. Finally, the choice of an appropriate phage library expression system, library format, and length of the peptides requires further optimization.

During the past 30 years, predominantly Sanger sequencing was used to identify peptides from eluted phages. However, we found that the same sequences were enriched in combination with numerous different targets. The explanation lies in false positive binders, including target-unrelated peptides and rapidly propagating phages. To avoid this problem, we recently employed next-generation sequencing (NGS) to evaluate whether a sequence was identified due to an amplification advantage of the phage or due to a true interaction with the target.

## Peptide characterization and production

Overall, biosorption can be categorized into two categories: physisorption refers to weaker, long-range ($\varDelta H < 40{kJ}.{mo}{l}^{-1}$) interactions, while chemisorption involves stronger, short-range interactions ($\varDelta H > 40{kJ}.{mo}{l}^{-1}$, up to several hundred ${kJ}.{mo}{l}^{-1}$). The underlying mechanisms of interaction between peptides and their target material can include Van-der-Waals forces, electrostatic interactions, hydrogen bonds, and hydrophobic interactions (Schwaminger et al, 2018).

During biopanning, a randomized pool of peptides attached to a bacteriophage surface interacts with the target material. After identification of strong-target-binding peptides, we characterize the interaction mechanisms with various tools, such as quartz crystal microbalance, isothermal titration calorimetry and vibrational infrared or Raman spectroscopy.

To conduct further tests, promising peptides are typically produced in low amounts via chemical solid-phase peptide synthesis. Here, we find that the majority of peptides identified via PSD do not bind to the target as effectively as their phage-attached counterparts; merely one out of ten synthesized peptides exhibit satisfactory interaction. These peptide candidates can be produced in higher amounts biologically. Further immobilization of biologically produced peptides onto a solid phase has the advantage of easy reuse of these biomolecules.

## Peptide applications for recycling critical raw materials from e-waste

Peptide-based recycling of critical raw materials from e-waste can be achieved largely through two approaches: the selective enrichment of metal-containing particles or the complexation of metal ions after leaching from solid materials. Here, we illustrate both approaches with research conducted by the BioKollekt group using Europium as a case study. Europium, a red fluorescent heavy REE, is predominantly used in artificial lighting products, notably as Y_2_O_3_:Eu^3+^ phosphor-powder in fluorescent lamps. Unlike most REEs, europium exhibits a unique ability to exist in the +II oxidation state alongside the common +III state. However, under natural aqueous conditions, europium ions are typically present as Eu^3+^.

**Figure 1:**
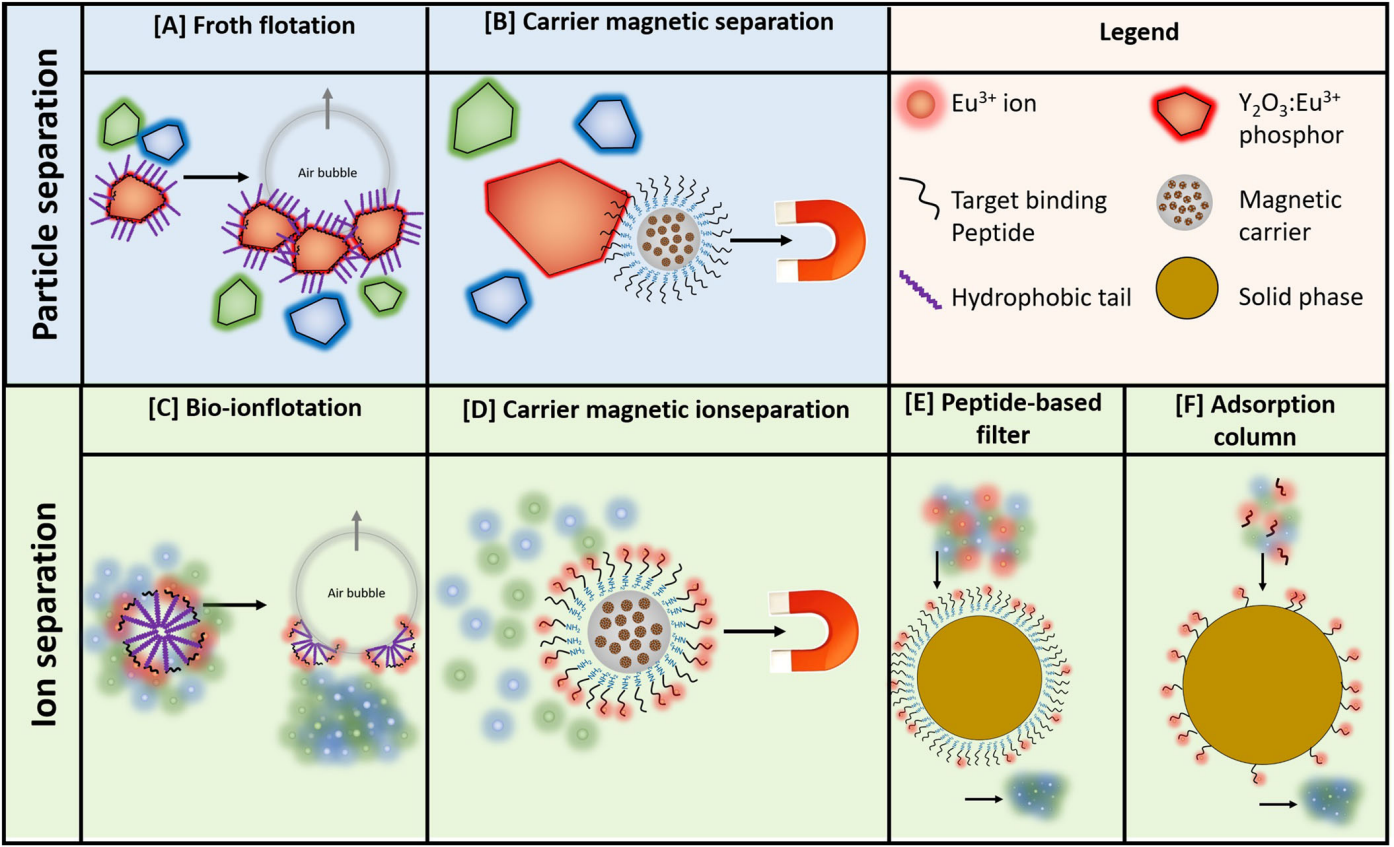
Conceptual overview of separation methods that use peptides for the purification of critical raw materials. For particles: (**A**) froth flotation and (**B**) carrier magnetic separation. For metal ions: (**C**) bio-ionflotation, (**D**) carrier magnetic ion separation, (**E**) peptide-based filter, and (**F**) adsorption column.

The BioKollekt group recently identified Eu^3+^-binding peptides using PSD (Techert et al, 2025). The Eu^3+^ ions were complexed with the highest affinity by peptides that express two or more acidic amino acids—glutamic or aspartic acid—and the role of other amino acids in the peptide sequence was investigated in detail. In a different approach, peptide-functionalized composite magnetic beads were tested for the separation of Y_2_O_3_:Eu^3+^ powder from mixtures containing other fluorescent phosphors (Boelens et al, 2022). The results revealed that the interaction between Y_2_O_3_:Eu^3+^ and the carrier is primarily governed by surface charges, enabling the purification of the red phosphor through electrostatic interactions with negatively charged carriers. Figure 1 provides an overview of the separation concepts in which target-binding peptides can be employed to recover metals as either particles or ions.

One major challenge for e-waste recycling is the much more complex composition of the secondary waste raw material compared to natural ores. To address with this problem, the PSD tool can find uniquely selective target-binding peptides by testing approximately one billion sequences in a single run. The thus obtained highly selective peptides can then be employed for e-waste recycling with conventional processes. For instance, flotation is one of the main separation processes used in mineral processing. By linking a hydrophobic tail to a peptidic head, a surfactant is obtained that serves as a highly selective collector in bioflotation (Fig. 1A). This process is relatively easily upscalable, since it is compatible with conventional flotation equipment. A linker between the surfactant’s tail and head can avoid a blocking effect of the hydrophobic tail on the interaction of the peptide with the target.

**Figure 2:**
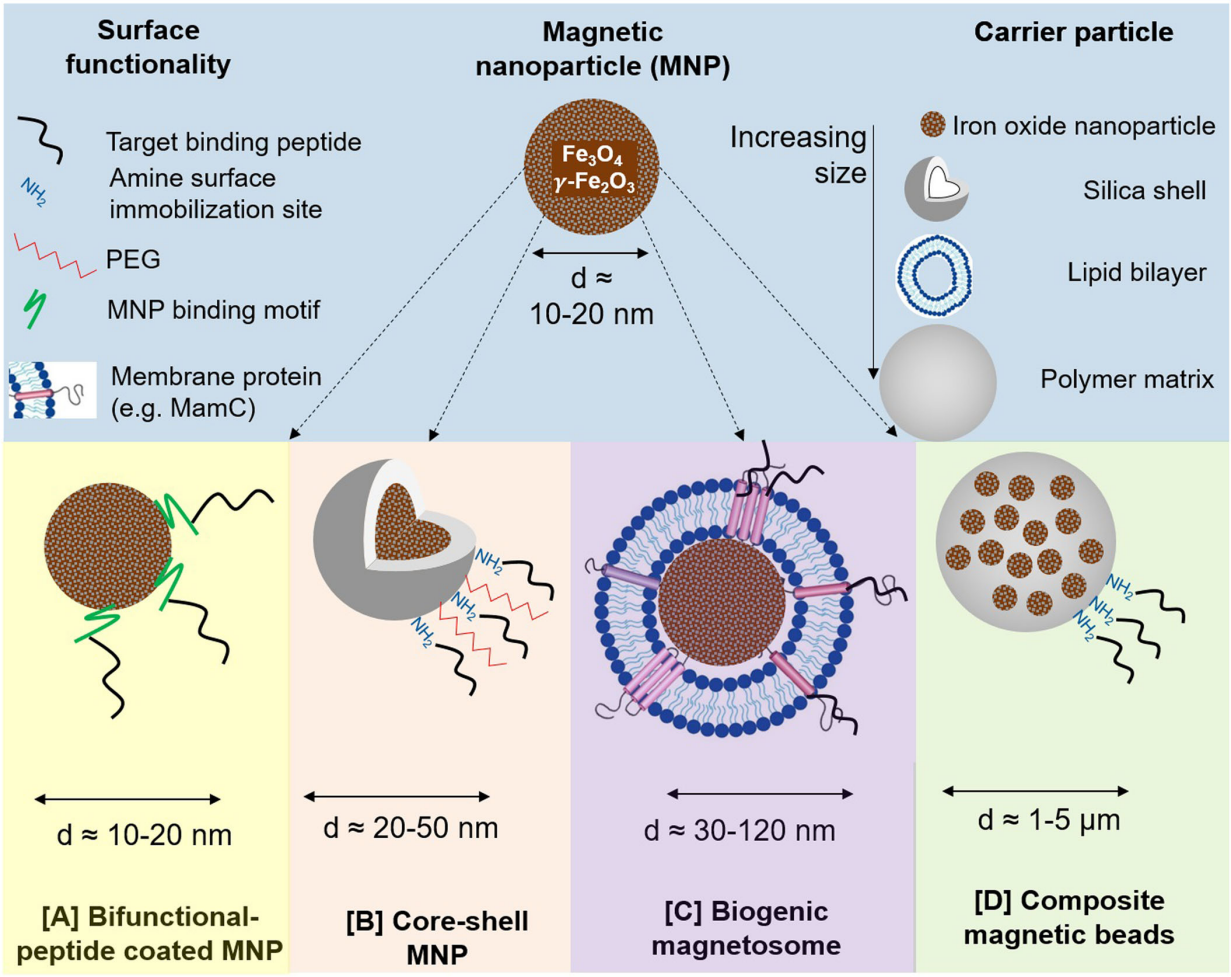
Overview of peptide-functionalizable magnetic carriers and their surface functionalities. (**A**) biofunctional-peptide-coated MNP, (**B**) core-shell MNP, (**C**) biogenic magnetosomes and (**D**) composite magnetic beads. Image (**C**) is adapted from Strbak et al, 2022.

Another challenge of e-waste recycling is the separation of very fine synthetic particles compared to comminuted natural ore bodies. Flotation is typically suitable for minerals with a particle size in the range of 50–250 µm. In the case of ultrafine particles with a size of just a few microns, the inferior contact between the target particle and air bubbles hinders efficient flotation. As a solution, peptides can be immobilized on magnetic carriers, which are able to efficiently separate fine target particles in an inhomogeneous magnetic field (Fig. 1B). In fact, the increasing specific surface area of particles with a decreasing size is expected to enhance separation efficiency with magnetic carriers. An additional advantage of immobilizing the peptides onto a carrier is that the peptides can easily be recovered and reused. Suitable magnetic carriers can include peptide-coated magnetic particles (MNP), composite magnetic beads, core-shell MNP and even magnetic cell organelles, so-called magnetosomes, produced by magnetotactic bacteria (Fig. 2).

Purification of metal ions can also be achieved with bio-ionflotation (Fig. 1C) and carrier magnetic separation (Fig. 1D). In bio-ionflotation, hydrophobized target-binding peptides form micelles, which are removed by rising air bubbles. Furthermore, separation of metal ions can also be achieved by solid-liquid separation either by immobilizing the peptides onto a solid phase (peptide-based filter (Fig. 1E)) or by using a solid phase that adsorbs the ion-peptide complex (adsorption column (Fig. 1F)).

Generally, the use of peptides in e-waste recycling has great potential to increase the recovery rates of critical raw materials. To reduce the costs for e-waste recyclers, these novel separation approaches need to be adapted to existing instruments such as flotation tanks and magnetic separators. Further advances and research by our group in the coming years will show whether the application of siderophores, biosurfactants, and peptides can achieve industrial scale in recycling.

“To reduce the costs for e-waste recyclers, these novel separation approaches need to be adapted to existing instruments such as flotation tanks and magnetic separators.”

## Conclusion

The increasing global demand for metals and REE, the need to transition to a circular sustainable economy along with EU laws to increase recycling quotas are putting pressure on the recycling and electronics industries to improve the recovery of essential elements from electronic waste. Novel recycling technologies that utilize biomolecules with high-affinity and specificity for target materials are therefore attracting growing attention. Peptides, as environmentally friendly biomolecules, can be sustainably produced from renewable plant-based resources, offer robust performance under industrial conditions, are non-toxic and biodegradable. Moreover, peptides could easily be used in well-established, existing industrial recycling processes even for elements and materials that challenge conventional processes. Employing these highly selective, high-affinity biomolecules could hopefully enable the industry to further increase recovery rates and meet or exceed legally prescribed quotas in a more environment-friendly way. As such, it would be a big step for a major industry towards a circular economy.

“Employing these highly selective, high-affinity biomolecules could hopefully enable industry to further increase recovery rates and meet or exceed legally prescribed quotas in a more environment-friendly way.”

## Supplementary information

Peer Review File
