# Supplementary material for: Peptide-based recycling of critical raw materials from electronic waste: The transition towards a circular economy requires novel and environmentally-friendly solutions to increase the recycling rate from discarded electronic devices
Source: EMBO Rep. 2025 Apr 23;26(9):2221–6. doi: 10.1038/s44319-025-00449-x (PMC12069698; doi:10.1038/s44319-025-00449-x)
Supplement: Supplementary file 1 — Peer Review File [file 44319_2025_449_MOESM1_ESM.pdf]

EMBO reports

Peptide-based recycling of critical raw materials from electronic waste

Franziska Lederer and Peter Boelens

Corresponding author(s): Franziska Lederer (f.lederer@hzdr.de)

Review Timeline:

Submission Date:	28th Mar 25
Accepted:	7th Apr 25

Editor: Holger Breithaupt

Transaction Report: This article was editorially reviewed.
